# An approach for jatropha improvement using pleiotropic QTLs regulating plant growth and seed yield

**DOI:** 10.1186/1754-6834-5-42

**Published:** 2012-06-15

**Authors:** Fei Sun, Peng Liu, Jian Ye, Loong Chuan Lo, Suying Cao, Lei Li, Gen Hua Yue, Chun Ming Wang

**Affiliations:** 1 Molecular Population Genetics Group, Temasek Life Sciences Laboratory, 1 Research Link, National University of Singapore, 117604, Singapore, Singapore; 2 Temasek Life Sciences Laboratory, 1 Research Link, National University of Singapore, Singapore, Singapore

## Abstract

**Background:**

Higher seed yield is one of the objectives of jatropha breeding. However, genetic analysis of the yield traits has not been done in jatropha. Quantitative trait loci (QTL) mapping was conducted to identify genetic factors controlling growth and seed yield in jatropha, a promising biofuel crop.

**Results:**

A linkage map was constructed consisting of 105 SSR (simple sequence repeat) markers converged into 11 linkage groups. With this map, we identified a total of 28 QTLs for 11 growth and seed traits using a population of 296 backcrossing jatropha trees. Two QTLs *qTSW-5* and *qTSW-7* controlling seed yield were mapped on LGs 5 and 7 respectively, where two QTL clusters controlling yield related traits were detected harboring five and four QTLs respectively. These two QTL clusters were critical with pleiotropic roles in regulating plant growth and seed yield. Positive additive effects of the two QTLs indicated higher values for the traits conferred by the alleles from *J. curcas*, while negative additive effects of the five QTLs on LG6, controlling plant height, branch number (in the 4th and 10th months post seed germination), female flower number and fruit number respectively, indicated higher values conferred by the alleles from *J. integerrima*. Therefore favored alleles from both the parents could be expected to be integrated into elite jatropha plant by further backcrossing and marker assisted selection. Efficient ways to improve the seed yield by applying the two QTL clusters are discussed.

**Conclusion:**

This study is the first report on genetic analysis of growth and seed traits with molecular markers in jatropha. An approach for jatropha improvement is discussed using pleiotropic QTLs, which will be likely to lead to initiation of molecular breeding in jatropha by integrating more markers in the QTL regions.

## Background

Jatropha seed contains about 18-30% oil, which can be pressed to produce high-quality biodiesel fuel. It is believed that jatropha can be used to alleviate the energy crisis, and is becoming one of the world’s key crops for biodiesel production [1,2]. However, jatropha must not directly compete with food crops due to possible food crisis and limited farmlands in the world, so it must be resistant to a high degree of aridity. Jatropha can therefore be used to rehabilitate wastelands and improve the environment. It can also enhance the quality of rural life by providing new economic resources for marginal farmlands [3].

Since jatropha has been domesticated, there is an immediate need to breed for superior genotypes. The objectives of breeding should aim at higher seed yield and oil content, earlier maturity, reduced plant height, resistance to pests and diseases, drought resistance/tolerance, higher ratio of female to male flowers and improved fuel properties [4]. Traditional methods of genetic improvement of quantitative traits have relied mainly on phenotype and pedigree information [5], which are easily influenced by environmental factors. Jatropha seed yield is a complex trait, with difficulties in reliable yield prediction. In order to tackle this knowledge gap, it is necessary to systematically study the annual seed yield in operational plantation conditions along with relevant factors [6].

As in other crops, almost all of the economically important traits in jatropha, such as seed yield, biotic or abiotic stress resistance, are quantitative and determined by multiple genes with minor effects which are described as quantitative trait loci (QTL). Taking rice for example, a number of genes (or QTLs) for yield traits, including tillering [7], number of grains per panicle [8] and grain weight [9], have been isolated through map based cloning. The genes regulating yield traits and developmental processes can function at various stages, in different pathways and through diverse mechanisms in rice [10]. A genomic region on chromosome 4 was detected with multiple effects on increased flag leaf width and length, and panicle number and length in rice [11]. Clustered QTLs were recently reported for source leaf size and yield traits in rice [12], and improving rice yield and quality by QTL pyramiding was carried out [13]. Marker assisted breeding has been applied by taking advantages of the useful information on these genes or QTLs affecting agronomic traits of importance.

In contrast, jatropha, which was still considered wild in 2010 [6], had not yet undergone a careful breeding program with systematic selection and improvement of suitable germplasm. Recently, we have established a first generation genetic linkage map using 506 microsatellite and SNP (Single Nucleotide Polymorphism) markers covering 11 linkage groups [14], and conducted a whole genome scan for QTL and eQTL affecting seed oil traits [15]. In addition, we have isolated and identified miRNAs and targets in jatropha [16]. However, the genetic bases of jatropha growth and seed yield have not been studied in jatropha breeding.

In this paper, we describe the genetic bases of seed yield through QTL mapping, which is one of the most important agronomic traits, together with plant height, stem diameter, branch number, female flower number and fruit number. Moreover we have analyzed the pleiotropic effects and interaction of the QTLs, and provided an approach for possible modulation of the QTLs to improve growth and seed character in jatropha.

## Results

### Trait analysis

Growth and seed traits were measured in a QTL mapping population, and the frequency distributions of all traits in the progeny showed a continuous distribution. The distribution of phenotypic values showed bi-directional transgressive segregation (Table 1), revealing complex genetic bases of these traits. While seed yield in *J. curcas* was higher than that in *J. integerrima*, branch number in *J. integerrima* is significantly higher than that in *J. curcas*. The data implied that *J. integerrima* germplasm could be applied for hybrid breeding to improve agronomic traits, such as branch number in the fourth and tenth months, and female flower number.

**Table 1:** **Descriptive statistics on phenotype data of QTL mapping population and parents (*J. curcas*PZMD16,*J. integerrima*S001 and F1 CI7041)**

**N**	**Trait**	**Acronym**	**Mean**	**SD**	**Min**	**Max**	**PZMD16**	**CI7041**
	**Growth traits**							
1	Height in the 4th month	H4M	70.5	24.6	15.0	134.0	62.16	70
2	Height in the 10th month	H10M	122.9	37.2	33.0	272.0	166	180
3	Diameter in the 4th month	D4M	1.9	0.5	0.6	3.2	1.45	1.2
4	Diameter in the 10th month	D10M	4.3	1.1	1.1	7.0	6.2	5
5	Branch number in the 4th month	BN4M	1.8	2.2	0.0	14.0	10	12
6	Branch number in the 10th month	BN10M	6.4	4.0	1.0	19.0	15	17
7	Total branch number	TBN	4.4	2.1	1.0	14.0	3.5	3.33
8	New branch number per branch	BNPB	2.9	1.1	1.0	8.0	1.75	1.67
	**Flower, fruit and seed yield**							
9	Female flower number	FFN	4.5	2.8	0.0	15.0	4.75	9.25
10	Fruit number	FRUITNO	9.1	10.5	0.4	62.0	35	No fruit (Hybrid F1)
11	Total seed weight in 2010	TSW	34.1	60.1	0.5	541.4	360.0	90.0

Correlation analysis among these traits was performed (Table 2), and total seed weight showed a significant correlation with total branch number, female flower number and fruit number, with coefficients 0.364, 0.294 and 0.308, respectively. Therefore, these agronomic traits were suggested to be key factors for seed yields.

**Table 2:** Correlation coefficients and significance of correlations among growth and yield traits in a QTL mapping population

	**H4m**	**H10m**	**D4m**	**D10m**	**BN4m**	**BN10m**	**TBN**	**BNPB**	**FFN**	**FruitNo**
H10m	0.371***									
D4m	0.652***	0.374***								
D10m	0.171***	0.675***	0.494***							
BN4m	0.170***	0.064	0.229***	0.177***						
BN10m	0.199***	0.189***	0.175***	0.401***	0.415***					
TBN	−0.155*	0.041	−0.092	0.154*	0.067	0.006				
BNPB	−0.027	−0.015	−0.005	0.094	0.141	0.072	0.450***			
FFN	−0.448***	0.138*	−0.207***	0.258***	−0.042	−0.061	0.265***	0.097		
FruitNo	0.026	0.220***	0.077	0.256***	0.198**	0.327***	0.097	0.038	0.215**	
TSW	−0.137*	0.093	0.004	0.262***	0.156*	0.164**	0.364***	0.048	0.294***	0.308***

*P* values are as follows: * *P* < 0.10, ** *P* < 0.05, *** *P* < 0.01.

### QTL mapping

The linkage map consisting of 105 DNA markers and covering 643.8 cM of the genome, converged into 11 LGs (linkage groups) corresponding to 11 chromosome pairs in jatropha. The average distance between markers was 6.6 cM. Most of the LGs were consistent with those described previously [14].

**Figure 1:**
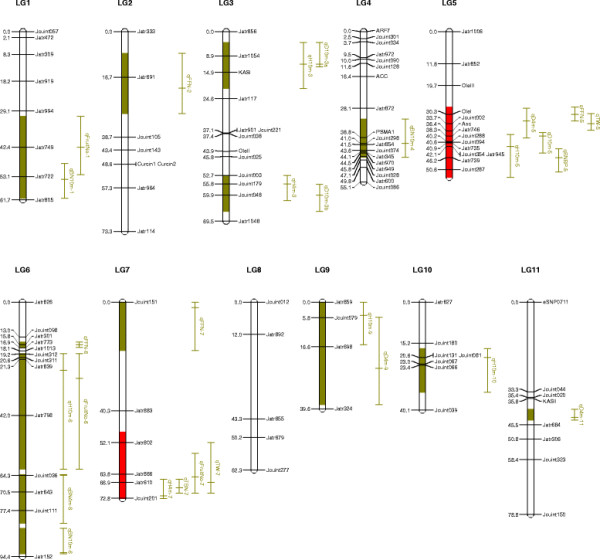
** Summary of QTL locations detected. QTL represented by bars are shown on the left of the linkage groups, close to their corresponding markers.** The lengths of the bars are proportional to the confidence intervals of the corresponding QTL in which the inner line indicates position of maximum LOD score. The confidence intervals of QTL are showed in green color, and two QTL clusters are highlighted in red.

QTL analyses were performed on the means of growth traits, branch number, female flower and fruit number, and seed yield (Table 3; Figure 1). We have detected 28 QTLs for all traits examined with LOD threshold 2.0 to 2.5 determined by permutations. Individual QTLs were detected with percentage of variation explained (PVE or R^2^) 3 to 21.16%, and four of them had PVE exceeding 10%.

**Table 3:** QTLs for growth traits, seed characters

	**Trait**	**QTL**^a^	**Linkage Group**	**Marker**	**Position**^**b**^**cM**	**LOD Peak**	**R**^**2 c (%)**^	**Additive Effects**^**d**^
Growth	H4m	*qH4m-3*	3	Jcuint179	55.8	3.50	5	−17.51
		*qH4m-7*	7	Jatr610	71.9	2.97	4.4	−10.31
	H10m	*qH10m-3*	3	Jatr1054	11.9	3.55	6.3	21.53
		*qH10m-5*	5	Jatr945	42.1	5.81	8.5	21.99
		*qH10m-6*	6	Jcuint312	25.3	2.56	4	−15.80
		*qH10m-9*	9	Jatr859	5.0	3.09	4.5	16.30
		*qH10m-10*	10	Jcuint081	20.6	4.63	6.7	−19.25
	D4m	*qD4m-5*	5	OleI	31.3	3.88	5.6	0.26
		*qD4m-9*	9	Jatr698	24.6	2.70	5.2	0.25
		*qD4m-11*	11	Jatr684	14.3	2.98	4.6	0.25
	D10m	*qD10m-3a*	3	Jatr1054	11.9	8.11	12.7	0.96
		*qD10m-3b*	3	Jcuint048	59.9	3.85	4.9	−0.68
		*qD10m-5*	5	Jatr746	38.3	15.03	21.1	1.11
	BN4m	*qBN4m-6*	6	Jcuint036	64.3	4.04	6.9	−1.26
	BN10m	*qBN10m-1*	1	Jatr722	54.1	3.44	5.6	2.04
		*qBN10m-4*	4	Jatr854	41.0	3.51	5.5	−2.19
		*qBN10m-6*	6	Jcuint111	93.4	3.58	5.9	−2.28
	TBN	*qTBN-7*	7	Jatr610	68.9	3.40	8.4	1.30
	BNPB	*qBNPB-5*	5	Jatr739	46.2	2.98	7.9	0.62
Flower, Fruit and seed yield	FFN	*qFFN-2*	2	Jatr691	20.7	2.00	4.5	1.20
		*qFFN-5*	5	OleI	30.3	3.38	7	1.56
		*qFFN-6*	6	Jatr301	15.8	6.41	13.6	−2.21
		*qFFN-7*	7	Jcuint151	2.0	4.16	9.6	1.97
	FruitNo	*qFruitNo-1*	1	Jatr749	42.4	2.47	5.5	5.54
		*qFruitNo-6*	6	Jatr839	28.3	4.97	12.4	−7.61
		*qFruitNo-7*	7	Jatr866	64.8	3.15	7.3	5.65
	TSW	*qTSW-5*	5	Jcuint002	33.7	2.24	5.2	29.33
		*qTSW-7*	7	Jatr866	66.8	2.70	4.9	26.31

^a^ QTL: starting with “q,” followed by an abbreviation of the trait name, the name of the linkage group, and the number of QTL affecting the trait on the linkage group. ^b^ Position from the first marker on each linkage group. ^c^ Coefficient of determination or the percentage of variance explained (PVE) by the detected QTL. ^d^ Estimated phenotypic effect of substituting *J. integerrima* alleles with *J. curcas* alleles at QTL.

QTLs with positive and negative additive effects were identified, with a positive effect implying a higher value for the trait conferred by the allele from *J. curcas*, and negative from *J. integerrima* (Table 3).

### QTLs for growth traits

Sixteen QTLs were identified and dispersed among all the linkage groups except LGs 2 and 8. Four QTLs overlapping on the lower part of LG5, namely *qH10m-5*, *qD4m-5*, *qD10m-5* and *qTBN-5*, were detected underlying plant height in the 10th month, stem diameter in the 4th and 10th months, and total branch number, respectively (Figure 1). Additive effects of these QTLs were positive, indicating that the alleles from *J. curcas* increased these trait values.

Conversely, two QTLs, namely *qBN4m-6* and *qBN10m-6*, were detected on the lower part of LG6 controlling branch number with negative additive values, indicating *Jatropha integerrima* allele increased branch number.

### QTLs for female flower and fruit number

Six QTLs were identified and dispersed on LGs 1, 5, 6 and 7, with two QTLs, namely *qFFN-6* and *FruitNo-6*, being located on the same region of LG6, controlling female flower number and fruit number respectively. The PVE of these two QTLs were higher than 10%, indicating their significant effects on the two important yield trait components.

### QTLs for seed traits

On LGs 5 and 7, two QTLs of *qTSW-5* and *qTSW-7* were detected controlling total seed weight, which is one of the most economically important traits. Interestingly, QTLs underlying yield related traits were clustered at these two QTLs. At *qWT-5*, four QTLs underlying plant height, stem diameter, branch number and female flower number were detected. Near *qTSW-7*, three QTLs of *qH4m-7*, *qTBN-7* and *qFruitNo-7* were detected, controlling plant height, total branch number and fruit number respectively.

**Figure 2:**
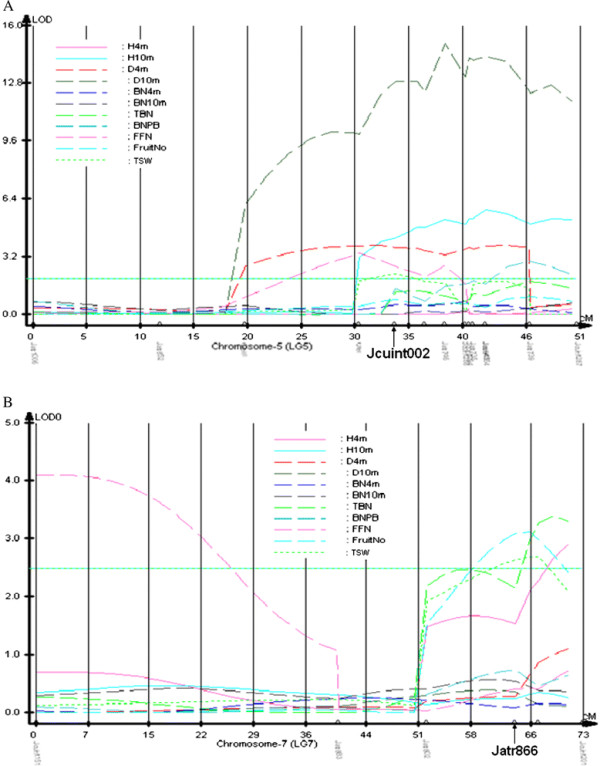
** QTL clusters on LGs 5 and 7.** QTL scans of growth on linkage maps. Horizontal line indicates 5% LOD significance thresholds (2.0) based on permutation. **A**: LG5; **B**: LG7.

It was noteworthy that two QTL clusters were detected on LGs 5 and 7, respectively. Five QTLs were detected on the lower part of LG5 (Figure 2A), and four QTL clusters were detected on lower part of LG7 (Figure 2B).

### Favored alleles originated from two parents

Two QTL clusters were detected consisting of five and four QTLs, controlling total seed weight, plant height, stem diameter, female flower number and fruit number. The positive additive effects indicated higher values for the traits conferred by the allele from *J. curcas*. Meanwhile five QTLs on LG6, namely *qH4m-6*, *qBN4m-6*, *qBN10m-6 qFFN-6* and *qFruitNo-6*, controlling plant height, branch number (in 4th and 10th months post seed germination), female flower number and fruit number respectively, were detected with negative additive effects indicating higher values conferred by *J. integerrima* (Table 3).

### Major effects of *qTSW-5* and *qTSW-7*

**Figure 3:**
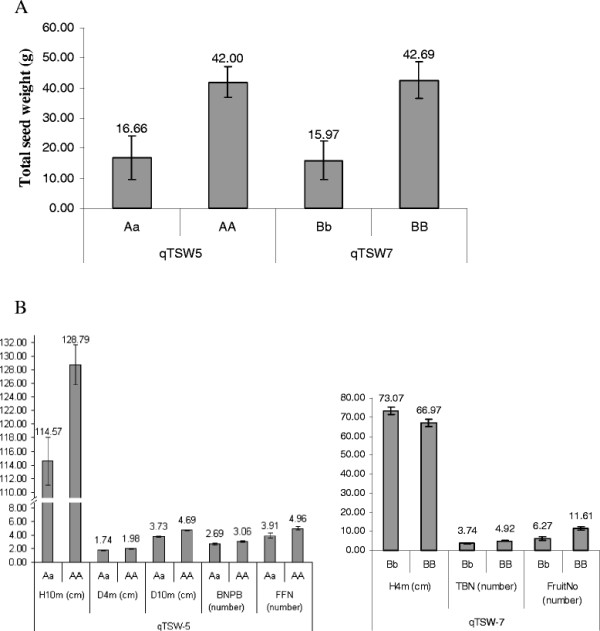
** Total seed weight (g) and related traits of plants with different genotypes of*qTSW-5*(AA, Aa) and*qTSW-7*(BB, Bb); N denotes sample number of each genotypic classes; Error bars denote SEs (Standard Errors).A**: Significant major effects of the two QTLs on seed yield; **B**: The two QTLs with pleiotropic roles in regulating plant growth and seed yield. Significant at P < 0.01 of Bonferroni test.

A two-way analysis of variance (ANOVA) was carried out to assess genetic effects and interactions of the two QTLs of *qTSW-5* and *qTSW-7* controlling total seed weight. The values of different genotypes are shown in Figure 3. Total seed weight was significantly increased in the presence of these two QTLs. When *qTSW-5* presented, total seed weight was improved from 16.66 ± 7.26 to 42.00 ± 5.06 g, and *qTSW-7*, from 15.97 ± 6.36 to 42.69 ± 6.16 g (Figure 3A).

Interestingly, we found that the two QTLs for seed yield overlapped with other QTLs for other agronomic traits than seed yield itself. ANOVA showed that the QTL *qTSW-5* for seed yield affected significantly plant height, stem diameter, new branch number per branch and female flower number, while *qTSW-7* affected plant height, total branch number and fruit number (Figure 3B).

### Effect of pyramiding *qTSW-5* and *qTSW-7*

**Figure 4:**
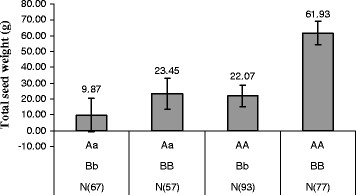
** Effects of pyramiding the two QTLs of*qTSW-5*(genotypes of AA, Aa) and*qTSW-7*(BB, Bb) on seed yield.** Error bars denote SEs.

The interaction between marker effects for *qTSW-5* and *qTSW-7* was non-significant with a relatively low P value (0.14) (Table 4), while the marker effects for *qTSW-5* and *qTSW-7* were non-additive (Figure 4). This could be caused by the lack of power in the ANOVA due to an unequal distribution of genotypic classes (Figure 3).

**Table 4:** ANOVA of seed yield in the QTL mapping population based on genotypes of the marker loci that are most closely linked to the QTLs

**Effect**	**d.f.**	**MS**	**F**	**P**
1 (Jcuint002, *qTSW-5*)	1	24943.135	8.190	0.005**
2 (Jatr866, *qTSW-7*)	1	27739.644	9.110	0.003**
1 X 2	1	6703.480	2.200	0.140

** Denotes significant at P = 0.01.

Despite the non-significance of the interaction of the two QTLs, total seed weight was significantly increased in the presence of the two QTLs. Lines carrying both QTLs produced an average 61.93 ± 7.31 g of seeds, nearly three times as much as any other genotype combinations (Figure 4). Therefore, although total seed weight could be improved by introducing the two QTLs, there would be advantages to be gained by pyramiding the two QTLs.

## Discussion

### Pleiotropic QTLs for growth and seed yield

Improvement of yield potential is the most important goal of most breeding programs worldwide. However, yield is a complex trait controlled by many genes with major or minor effect [17]. Till date, selective breeding for high yield remains the most challenging task. Our results show that QTL clusters may have multiple effects on yield and yield-related traits, and we have detected two QTL clusters controlling multiple traits on the same regions of LGs 5 and 7. The two QTLs for seed yield clustered with those for plant height, stem diameter, branch number, female flower number or fruit number, revealing that these are two critical regions for jatropha growth and seed yield. Similarly, a major QTL, *Ghd8*, plays pleiotropic roles in regulating grain productivity, plant height, and heading date in rice [18]. These yield-related traits, such as height, diameter, branch number, female flower number, fruit number and so on, are less complex than total seed yield *per se*, yet highly correlated with total seed yield, hence it could be very useful for selection at different breeding stages for evaluating respective agronomic traits [19].

Some of the pleiotropy could be a consequence of correlations among traits such as female flower number, fruit number and seed yield, or between diameter and height, which were highly correlated at the phenotypic level. Some of the co-occurrence of QTLs could be a natural consequence of allometry, as has been suggested in the study on poplar [20]. In spite of that, the allometric relationship may not exist in alternative genetic backgrounds that have been exposed to different selection pressures [20].

The pleiotropic QTLs could be explained in different ways. Chromosomal regions were associated with more than two traits indicating either linkage or pleiotropic effects. There could be certain genes coexisting in these QTLs or a certain gene with pleiotropic effects on jatropha growth and seed development. Here, the QTLs we reported were still distant to the flanking markers with linkage distance, therefore, it will be meaningful to conduct fine mapping of these QTLs, isolate the target genes, and understand whether linkage or pleiotropic effects are responsible. Fine mapped QTL will speed up genetic improvement through marker assisted selection (MAS) by applying the closely linked markers [21]. As molecular markers are still limited in jatropha, we are constructing a second generation linkage map of jatropha with a high-resolution of SSR or SNP markers, which will lay a solid foundation for a variety of future genetic and genomic studies, including QTL fine mapping and marker assisted breeding.

### Towards molecular breeding by transferring favored alleles from the two parents

Plant growth and seed traits in jatropha are controlled by multiple gene complexes. Genetic markers have made it possible to detect QTLs that are significantly associated with traits, making selection more effective [21]. Genetic response can be further improved by inclusion of the QTLs in marker assisted breeding, which makes use of phenotypic, genotypic and pedigree data [22]. DNA markers have enormous potential to improve via MAS the efficiency and precision of conventional plant breeding [23], including jatropha germplasm enhancement and genetic improvement. The exploitation of the advantages of MAS relative to conventional breeding could have a great impact on crop improvement. We have identified markers linked to some major QTLs and genes by constructing a backcross population between *J. curcas* and *J. integerimma*. Further interspecific introgressions could be expected to apply the allelic sources for trait improvement.

The QTLs of *qTSW-5* and *qTSW-7* controlling seed yield were detected in two QTL clusters on LGs 5 and 7 respectively. The positive additive effects indicated higher values for the traits conferred by the alleles from *J. curcas*. Meanwhile the five QTLs on LG6, controlling plant height, branch number (in the 4th and 10th months post seed germination), female flower number and fruit number, were detected with negative additive effects, indicating higher values conferred by *J. integerrima*. Therefore, it will be feasible to transfer favored alleles from both the parents to elite jatropha varieties as recurrent lines. Hence, the QTL mapping population will be very useful in transferring favored alleles from both the parents by further backcrossing and marker assisted breeding.

### QTL pyramiding

Accumulating major genes for seed yield in an elite genotype by conventional breeding is laborious and time-consuming [24]. Gene pyramiding is difficult using conventional phenotyping methods, due to the epistatic effects of genes [25]. However, an advantage of pyramiding the two QTLs governing seed yield was observed in our study, and the identification of markers linked with each QTL allows for the identification of plants carrying one or both QTLs. In rice, rice yield and quality were improved by QTL pyramiding. The pyramid line (qHD8 + GS3) had higher yield potential, longer grains, and a more suitable heading date [13]. Our results reveal that detailed information regarding the pyramiding effect is very important for efficiency of marker assisted pyramiding of different alleles at target QTL in jatropha breeding. The markers described here may serve as useful tools for gene pyramiding with the two QTLs. Advanced lines with a good genetic background and high seed yield genes combinations will be expected to have great practical breeding value. Nevertheless, two issues need to be further addressed, one is whether effects of the QTLs are family specific; another is whether these QTLs will be robust enough to apply to other environments. Therefore the markers need to be further tested in different families and environments.

## Conclusions

We identified a total of 28 QTLs underlying the growth and seed yield traits in jatropha. This study represented the first investigation on plant growth and seed yield through QTL mapping in jatropha. An approach was discussed for jatropha improvement using pleiotropic QTLs, which could be likely to lead to initiation of MAS by integrating more markers in the critical regions of the two QTL clusters.

## Methods

### Plant material and growth conditions

*J. curcas* PZMD16 was crossed to *J. integerrima* S001 and hybrids F1 lines were generated. Then a BC1F1 population was constructed consisting of 296 individuals derived from the backcross between PZMD16 used as recurrent parent line and an F1 line named as CI7041. The population and parental lines were planted under standard growth conditions with spacing 2 m X 2 m in 2008 in experimental field of Lim Chu Kang farm, Singapore. Due to jatropha’s perennial life cycle, we generated backcross populations in 2008 and observed phenotypes from 2009.

We used the fertilizers 15-15-15(N-15%, P2O5-15%, K2O-15%) and 13-13-21(N-13%, P2O5-13%, K2O-21%) to promote growth of flowers and fruits. The fertilizer application was three times per year. In 2008, we applied 100 g of each fertilizer per plant each time. And we applied 200 g of each fertilizer per plant each time from 2009. The insecticides Rogor L-40 (Dimethoate 38% w/w) or Alcalineum (Mineral 80% w/w) were used in the farm every two months with the concentration of 3-5 ml/10 L. The fungicides were Mancozide WP (Mancozeb 80% w/w) or SAPROL(Triforine 17.8% w/w), applied once every two months with the concentration of 22 g/10 L water or 12.5 ml/10 L water, respectively.

### DNA markers and genotyping

We extracted total DNA from leaves using the DNeasy plant mini kit (QIAGEN, Germany). We selected one hundred and five markers almost evenly covering the 11 LGs from a first-generation linkage map of jatropha [14]. One primer of the selected markers was labeled with FAM or HEX fluorescent dyes at the 5' end. The PCR for microsatellite amplifications on PTC-100 PCR machines (MJ Research, CA, USA) was done using the program as follows: 94 °C for 2 min followed by 37 cycles of 94 °C for 30 s, 55 °C for 30 s and 72 °C for 45 s, then a final step of 72 °C for 5 min. Each PCR reaction consisted of 1× PCR buffer (Finnzymes, Espoo, Finland) with 1.5 mM MgCl_2_, 200 nM of each PCR primer, 50 μM of each dNTP, 10 ng genomic DNA and one unit of DNA-polymerase (Finnzymes, Espoo, Finland). Products were analyzed using a DNA sequencer ABI3730xl (Applied Biosystems, CA, USA), and fragment sizes were determined against the size standard ROX-500 (Applied Biosystems, CA, USA) with software GeneMapper V4.1 (Applied Biosystems, CA, USA) as described previously [26].

### Agronomic trait measurement and data collection

The growth traits were observed in the 4th and 10th months post seed germination because most of the plants started flowering in the 4th month and completed growth in the 10th month. We decided to observe the growth traits in the two critical times. Phenotypic data were collected from the QTL mapping population as follows:

Growth traits included height, diameter, branch number in the 4th and 10th months, total branch number and new branch number per pruned branch. Pruning is an agronomic treatment to produce more branches.

Flower and fruit traits included female flower number and fruit number, which were observed in three flower and fruit clusters respectively. The average numbers of the three replications were used for further QTL analysis.

In 2010, we harvested the fruits of each plant with separate nylon mesh bags twice a month and dried them in the sun. Then we hulled them to get the seeds. After recording the seeds’ number and dry weight, we stored them inside a 4 °C cooling room. All the seeds harvested in 2010 were collected for evaluating total seed weight of one year. Seeds were harvested at maturity after the color of the fruits had changed from green to yellow-brown.

### Statistical analysis

Plant height, diameter and branch number traits were collected at different stages in the backcross population consisting of 296 individuals, and traits of flower and fruit were collected with three replications. Pearson phenotypic correlations were calculated among all the traits using SAS PROC CORR [27].

Linkage map was constructed using the software CRIMAP 3.0 [28] with the genotyping data of 105 markers in the QTL mapping population. Kosambi function was used to calculate all multipoint distances. Graphical visualization of the linkage groups was completed with MapChart 2.2 software [29]. QTL analysis was carried out using QTL Cartographer version 2.5 [30]. Composite interval mapping (CIM) was used for mapping QTLs and estimating their effects. The forward regression method was used to scan the genome at 2-cM intervals. The log of the odds (LOD) score was determined for declaring a significant QTL by permutation test analyses (1,000 permutations, 5% overall error level).

The position and confidence interval of QTL were determined as described previously [15]. Briefly, the maximum LOD score was taken as the position of the QTL, and the region in the LOD score within 1 LOD unit of maximum was taken as the confidence interval. Additive effects of the detected QTL were estimated as the mean effects of replacing hybrid (CI7041)’s alleles at the locus of interest by *J. curcas* (PZMD16) alleles. Thus, for a QTL to have a positive effect, the *J. curcas* alleles must increase the trait value. The contribution of each identified QTL to total phenotypic variance (R^2^) or percentage of variance explained by the QTL (PVE) was estimated by variance component analysis. QTL nomenclature was adapted as follows: starting with “q,” followed by an abbreviation of the trait name, the name of the linkage group and the number of QTL affecting the trait on the linkage group.

Using marker genotypes as the groups, analysis of variance (ANOVA) was performed with the general linear model (GLM) procedure of SAS [27].

## Abbreviations

ANOVA, Analysis of variance; GLM, General linear model; LG, Linkage group; MAS, Marker assisted selection; QTL, Quantitative trait loci; SE, Standard error; SNP, Single nucleotide polymorphism; SSR, Simple sequence repeat.

## Competing interests

The authors declare that they have no competing interests.

## Authors’ contribution

FS and PL performed the experiments for collecting genotype and phenotype data. CMW and GHY conceived of the experiments. CMW analyzed the data and wrote the manuscript. GHY supervised the project on jatropha molecular breeding and revised the manuscript. JY, LCL, SYC and LL participated in laboratory and field work for data collection. JY conceived of the experiments and revised the manuscript. All authors read and approved the final manuscript.
